# Hybrid CNN-GRU Model for Real-Time Blood Glucose Forecasting: Enhancing IoT-Based Diabetes Management with AI

**DOI:** 10.3390/s24237670

**Published:** 2024-11-30

**Authors:** Reem Ibrahim Alkanhel, Hager Saleh, Ahmed Elaraby, Saleh Alharbi, Hela Elmannai, Saad Alaklabi, Saeed Hamood Alsamhi, Sherif Mostafa

**Affiliations:** 1 Department of Information Technology, College of Computer and Information Sciences, Princess Nourah Bint Abdulrahman University, P.O. Box 84428, Riyadh 11671, Saudi Arabia; 2 Faculty of Computers and Artificial Intelligence, Hurghada University, Hurghada 84511, Egypt; 3 Insight SFI Research Centre for Data Analytics, School of Engineering, University of Galway, University Road, H91 TK33 Galway, Ireland; 4 Research Development, Atlantic Technological University, Letterkenny, H91 AH5K Donegal, Ireland; 5 Department of Computer Science, Faculty of Computers and Information, South Valley University, Qena 83523, Egypt; 6 Department of Computer Science, College of Science and Humanities in Dawadmi, Shaqra University, Shaqra 11961, Saudi Arabia; 7 Department of Computer Science and Engineering, College of Informatics, Korea University, 145 Anam-ro, Seongbuk-gu, Seoul 02841, Republic of Korea; 8 Faculty of Engineering, IBB University, Ibb 70270, Yemen

**Keywords:** IoT-based diabetes, real-time blood glucose forecasting, convolutional neural networks, healthcare, blood sugar, hybrid model

## Abstract

For people with diabetes, controlling blood glucose level (BGL) is a significant issue since the disease affects how the body metabolizes food, which makes careful insulin regulation necessary. Patients have to manually check their blood sugar levels, which can be laborious and inaccurate. Many variables affect BGL changes, making accurate prediction challenging. To anticipate BGL many steps ahead, we propose a novel hybrid deep learning model framework based on Gated Recurrent Units (GRUs) and Convolutional Neural Networks (CNNs), which can be integrated into the Internet of Things (IoT)-enabled diabetes management systems, improving prediction accuracy and timeliness by allowing real-time data processing on edge devices. While the GRU layer records temporal relationships and sequence information, the CNN layer analyzes the incoming data to extract significant features. Using a publicly accessible type 1 diabetes dataset, the hybrid model’s performance is compared to that of the standalone Long Short-Term Memory (LSTM), CNN, and GRU models. The findings show that the hybrid CNN-GRU model performs better than the single models, indicating its potential to significantly improve real-time BGL forecasting in IoT-based diabetes management systems.

## 1. Introduction

Predicting glucose levels pose a significant challenge for numerous individuals with diabetes [1]. Diabetes is a chronic and irreversible medical condition characterized by the continuous elevation of BGL. The persistent rise is attributed to the body’s inefficiency in generating or properly utilizing insulin, which is responsible for regulating the blood sugar level. Forecasting BGL using DL has emerged as a promising approach to diabetes management to provide timely insights to patients [2]. These advancements promise to optimize insulin dosing, prevent hypoglycemic events, and ultimately enhance diabetes patients’ standard of life.

Type 1 diabetes is a long-term metabolic disorder [3] that, unfortunately, does not currently have a cure [4]. However, effective management strategies can significantly alleviate the signs and symptoms and decrease the likelihood of both the short-term and long-term consequences associated with the disease [5]. As a result, individuals with type 1 diabetes, as well as their caregivers, are typically provided with education on the established protocols for controlling the disease [6]. Managing type 1 diabetes through self-care can be challenging and susceptible to human errors. Therefore, the automation of diabetes management tasks would be highly advantageous. Significant progress has already been made in this area. For instance, technological advancements, including continuous glucose monitoring biosensors and insulin pumps, now benefit numerous individuals with type 1 diabetes [7]. Continuous glucose monitoring biosensors are minimally invasive devices that regularly capture blood glucose levels, aligning with the recommended practice of frequently monitoring glycemic status. Insulin pumps, on the other hand, require semi-automated insulin administration, requiring minimal user intervention. Furthermore, ongoing efforts are being made to develop entirely painless, constant BGL monitoring sensors, aiming to enhance the effectiveness of diabetes management [8].

Forecasting the BGL of individuals with type 1 diabetes is a complex task due to the highly unpredictable nature of this condition. As a result, DL has gained significant traction in blood glucose level prediction, following the trend observed in various other time-series forecasting domains [2,9]. Consequently, extensive research efforts have been dedicated to advancing the analysis techniques in this area. Despite the notable progress made thus far, challenges must be effectively addressed. The present work aims to contribute to addressing one such challenge [10].

This paper is motivated by the need to automate and increase the accuracy of BGL monitoring and forecasting based on real-time data processing, as well as improving patient quality of life and enabling tailored treatment [1]. The application side of diabetes management is intended to be improved. research gap This paper tackles the complexity of BGL prediction, an area where single models like CNNs, Long Short-Term Memory (LSTM), and GRUs are inadequate [2]. Combining the best features of both CNNs and GRUs, the suggested hybrid CNN-GRU model significantly increases prediction accuracy by capturing intricate temporal correlations in BGL data. Furthermore, this study used a publicly accessible type 1 diabetes dataset to test the hybrid model against existing approaches, and it addresses the need for optimal model architecture selection by utilizing a Bayesian optimizer [11]. The main goal of this paper is to propose optimized hybrid models to forecast BGL in different forecasting scenarios. Hybrid models based on CNN and DL models extract the deep features of the BGL time series and enhance the performance of the models.

The main contributions of this paper are as follows:

- We propose a hybrid (CNN-GRU) Convolutional Neural Network and Gated Recurrent Unit model for the multi-step-ahead forecasting of BGL. Combining CNNs with GRUs allows the model to extract meaningful features from the input data using the CNN layers and then use the GRU layers to capture temporal dependencies and sequence information based on the features extracted from real-time data coming from IoT-based diabetes management systems.
- We apply a Bayesian optimizer to select the best architectures of different models: CNN, LSTM, GRU, and hybrid models. The proposed approach is well suited to be incorporated into the Internet of Things (IoT) frameworks, as sophisticated optimization approaches are used to enhance its prediction performance. The results show that the hybrid technique can provide accurate and timely forecasts, which are essential for managing diabetes effectively.
- We evaluate model performance using a dataset comprising real-time blood glucose values from a single patient with type 1 diabetes using different lags and different BGL forecasting steps ahead. The proposed model outperformed all other models using different evaluation metrics and in different forecasting scenarios.
- CNN-GRU was integrated with big data platforms (Spark and Kafka) to forecast BGL in real-time and test its incorporation with cutting-edge technologies, such as Spark and Kafka, for establishing real-time monitoring systems. This integration enables seamless data collection and analysis, facilitating timely prediction and intervention.

This paper’s remaining sections are organized as follows: Section 2 presents relevant work. Section 3 introduces the BGL forecasting steps. Section 4 discusses the experimental findings. Lastly, Section 5 provides the conclusions.

## 2. Related Work

In this section, several recent publications on BGL forecasting are concisely summarized. To better align with the focus of this work, the following overview emphasizes the application of cutting-edge ML techniques and the utilization of type 1 diabetes datasets for the development and evaluation of models [2,12]. A recent study explored a multitask approach to forecasting BGL using the Ohio datasets [11]. The idea of transfer learning served as a basis for the models. The main objective was to address the issue of having a lot of personalized data to arrive at an accurate blood glucose prediction. To this end, the researchers first pre-trained a model on a source domain. They then trained a multitask model on the full dataset and leveraged these learned representations to construct personalized predictive models. The authors demonstrated their proposed approach’s efficacy by comparing its performance to subject-isolated and sequential transfer learning methods. A recent study introduced an autonomous channel setup for the DL-based prediction of BL [13]. Their proposed method adaptively selected history lengths for different variables, considering their time-dependency scales. The key idea was to avoid discarding valuable information from variables with a lasting influence while also preventing the inclusion of unhelpful data from variables with transient impacts. The models developed in this study were subjected to a comparative analysis against standard non-autonomous channel structures using both clinical and mathematical evaluation metrics. In [14], a DL approach employing a dilated Recurrent Neural Network (RNN), combined with transfer learning concepts, was introduced for blood glucose level prediction. The study focused on developing personalized models using the Ohio dataset for individuals with type 1 diabetes. Short-term forecasting tasks were employed to evaluate the proposed method. The outcomes indicated that this strategy surpassed conventional methods like a CNN, autoregressive models, and even support vector regression.

In [15], the authors proposed a univariate approach for predicting blood glucose levels. RNNs were employed as the learning models. The models were trained end-to-end to forecast future BGL at 30 and 60 min using only historical BG data as input; the developed models were evaluated using an Ohio dataset. The findings were equivalent to those of innovative studies run on the same dataset. The study examined the certainty of the predictions. To accomplish this, a parameterized multivariate Gaussian model was used to compute the standard deviation of the forecasts, which represented the forecast’s uncertainty. A study applied IOT ideas to evaluate four commonly used glycemic modeling approaches: support vector machine (SVM), Bayesian regularized neural network, multilayer perceptron, and Gaussian techniques that were [16]. used to evaluate the feasibility of mapping the complicated patterns within data on type 1 diabetes collected from 25 people. The findings demonstrated the possible role of such an analysis in improving diabetes management. Furthermore, among the examined approaches, the Bayesian regularized neural network model outperformed the others.

Nemat et al. [17] offered two techniques based on the data fusion of continuous glucose monitoring (CGM) and activity data utilizing stacked regression. Using CGM data records and activity data medians, the first technique trained three base regressions: MLP, LSTM, and Partial Least Squares Regression (PLSR). The second strategy used the same underlying regressions that had been trained once with activity windows and once with CGM data. Obeidat et al. [18] proposed ML models based on PLSR, Artificial Neural Networks (ANNs), K-Nearest Neighbors (KNN), and Decision Trees (DTs) for tracking patients’ blood sugar levels and predicting suitable insulin dosages. The results revealed that an ANN was the most reliable predictor of insulin patterns. A CNN for BGL prediction was proposed by El Idrissi et al. [19]. Recursive, Direct, Multiple-Input Multiple-Output (MIMO), Direct Recursive (DirRec), and Direct Multiple-Output (DirMO) were five multi-step-ahead forecasting (MSF) strategies used in the authors’ model to forecast a succession of values in a time series and determine the ideal setup for the suggested CNN. The Wilcoxon statistical test compared CNN and LSTM models using the DirecNet dataset.

Four data-driven models were developed by Zhang et al. [20] utilizing regression and deep learning to produce multi-horizon predictions in T1D patients. The Seq-to-Seq LSTM and MLR models performed well in forecasting BG levels within 30 min and 60 min. According to the results, the Seq-to-Seq LSTM and regression models can produce accurate predictions. The best outcomes were 17.52 and 10.91 for RMSE and MAE, respectively. Rabby et al. [21] put forward a strategy for forecasting BGL by employing a stacked LSTM-based RNN model that takes sensor malfunctions into account. The Kalman smoothing method was used to correct incorrect CGM data caused by sensor inaccuracy. The results were 6.45 and 17.24 mg/dL on average for prediction horizons (PHs) of 30 and 60 min.

Zhu et al. [22] developed a DL model based on an RNN and attention. The model was evaluated on an array of clinical datasets comprising 47 patients suffering from type 1 diabetes. The results proved that the proposed model could achieve superior results in terms of RMSE, MAE, and glucose RMSE, as well as recognizing hypoglycemia with the highest accuracy achievable. Li et al. [23] presented a deep neural network-based model that employs prior patient data, such as glucose levels, meal information, and insulin dosages, to predict the probability of developing type 1 diabetes in the future. The model is based on data preprocessing, followed by label transformation/recovery, and then multiple layers of a CNN. GluNet was examined using two clinical datasets, and the results showed that it obtained the best RMSE. Shahid et al. [24] developed a model that could forecast BGL based on a CNN and GRU. The presented model was able to predict BGL with the best RMSE.

### 2.1. Real-Time Data with IoT

In [25], the authors applied distributed ML on streaming data to predict disease in real-time. They used DTs, Spark, and Kafka to develop real-time systems. The DT method was applied to historical disease data, such as heart conditions and diabetes. Streaming data were sent to a Kafka topic, and then Spark Streaming read the data and sent them to DTs to predict health status. As demonstrated in the experiments, the proposed system can process and indicate massive amounts of distributed and medical data enabled by IoT in real-time.

Li et al. [23] presented DL model that employs prior patient data, such as glucose levels, meal information, and insulin dosages, to predict the probability of developing type 1 diabetes in the future; also, the Relief feature selection algorithm was used to select the best features. RF obtained the highest accuracy in predicting heart disease in real-time. Kafka read data from Twitter and sent it to Spark. Spark Streaming received data from Kafka and applied RF to predict heart disease in real-time.

In [26], the authors applied univariate and Relief feature selection methods with ML models (DT SVM, RF, and LR) to a heart disease dataset to obtain the best ML model for predicting heart disease in real-time. RF obtained the highest accuracy when evaluating the system in real-time. Kafka read data from Twitter and sent it to Spark. Spark Streaming received data from Kafka and applied RF to predict heart disease in real-time. In [27], the authors applied the DL models LSTM, BI-LSTM, and GRU to a historical BP time-series dataset to forecast BP. Near-future SBP was predicted in real-time using the BI-LSTM model, which delivered the best results in experiments. The simulated sensor was used to generate streaming BP and send it to a Kafka topic, and then Spark Streaming read the data streams from Kafka, applied a sliding window to the data, and sent them to BI-LSTM to predict BP soon.

### 2.2. Discussion of the Importance of Novel Approaches

Since traditional models frequently struggle with accuracy and flexibility, the growing complexity of managing diabetes, especially for those with type 1 diabetes (T1D), emphasizes the need for innovative blood glucose level (BGL) forecasting techniques. Current research highlights the shortcomings of conventional approaches and the possibility of improving forecasting accuracy using sophisticated machine-learning techniques, such as hybrid models that include CNNs and GRUs. Furthermore, tailored models that use real-time data from IoT devices are essential for customizing treatment to each patient’s unique profile. Adopting these cutting-edge tactics is crucial to improving patient outcomes and advancing diabetes treatment.

## 3. Methodology

This section covers the methodology for developing and evaluating BGL forecasting models and the steps of forecasting BGL in real-time.

**Figure 1:**
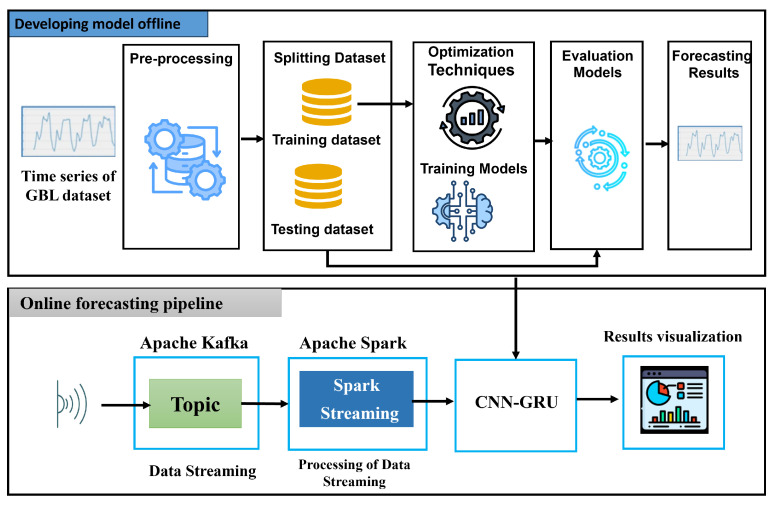
Stages of model development for blood glucose level monitoring.

Figure 1 shows the two main components: developing the model offline and the online forecasting pipelines. We describe each component in its own subsection.

### 3.1. Developing Model Offline

The offline model development procedures are depicted in Figure 1. In the ensuing subsections, each process is explained in depth. LSTM, GRU, CNN, and two hybrid models, CNN-LSTM and CNN-GRU, were used for predicting BGL using DL.

#### 3.1.1. Dataset Description

We used a BGL dataset [28] that contains a time series of blood glucose values from a single patient with type 1 diabetes. A sample of the BGL time-series dataset is shown in Table 1.

#### 3.1.2. Splitting Dataset

The BGL time-series dataset is split into a 75% training set and a 25% testing set, using different lags (3, 10, 13, 18, 20, and 30 min) to forecast BGL at different times in advance (5, 15, 20, 25, 30, and 60 min, respectively).

#### 3.1.3. Deep Learning Models

We forecast BGL using different DL models: LSTM, GRU, and CNN. The models are described in this section:

- The LSTM network computes long-term connections in a sequence of data time steps. The hidden layer of an LSTM network is made up of gated units. It comprises four layers that generate the cell’s state alongside its output. It then proceeds to the next hidden layer—three operational sigmoid gates and a tanh layer. Gates limit the amount of information transferred through the cell by determining which information is required by the next cell and which should be discarded. The network obtains data in time series or sequences from a sequence input layer [29]; an LSTM layer [29] creates long-term correlations across the time steps of sequential data. The figure depicts the progression of a time series X with S-length features over the LSTM layer. The input and forget gates control how data are gathered and placed in the cell state. Following the setting up of these two points, the cell state may be modified. The output gate then determines the ultimate output of the gating system. Equations (1)–(5) below determine the status of each node:
  (1) $$f_{t}=\sigma \left(W_{f}\cdot \left[h_{t-1},x_{t}\right]+b_{f}\right)$$
  (2) $$i_{t}=\sigma \left(W_{i}\cdot \left[h_{t-1},x_{t}\right]+b_{i}\right)$$
  (3) $${\overset{ˇ}{c}}_{t}=\tanh\left(W_{c}\cdot \left[h_{t-1},x_{t}\right]\right)+b_{c}$$
  (4) $$c_{t}=f_{t}*c_{t-1}+i_{t}*{\overset{ˇ}{c}}_{t}$$
  (5) $$o_{t}=\sigma \left(W_{0}\cdot \left[h_{t-1},x_{t}\right]+b_{0}\right)$$
  where $h_{t-1}$ represents the former layer’s hidden state, $x_{t}$ indicates the instant input, b and W denote the bias and weight, respectively, σ defines the sigmoid function, $f_{t}$ is the forget gate’s output, ${\overset{ˇ}{c}}_{t}$ denotes the output of the input gate, and $C_{t-1}$ stands for the moderate temporary state.
  From the preceding layer’s cell state, in the subsequent layer, $o_{t}$ is the output of the output gate, and $h_{t}$ is the following layer’s hidden state.
- The GRU’s architecture comprises a single hidden layer of recurrent neurons, each of which evaluates a given input sequence at a time step and generates a hidden state output [30]. Every neuron’s hidden state is updated via a succession of gating processes in response to the current input and the previous hidden state [30]. The GRU output is then computed using the updated hidden state. The update gate specifies which portions of the previously hidden state should be preserved and how much new information should be supplied. It is estimated as follows [31]:
  (6) $$z_{t}=\mathrm{sigmoid}\left(w_{z}*x_{t}+u_{z}*h_{\left\{t-1\right\}}+b_{z}\right)$$
  The reset gate decides how much of the previous hidden state to forget to make room for new data. It is calculated as follows:
  (7) $$r_{t}=\mathrm{sigmoid}\left(w_{r}*x_{t}+u_{r}*h_{\left\{t-1\right\}}+b_{r}\right)$$
  Here, x_t is the input at time step t, $h_{\left\{t-1\right\}}$ is the previous hidden state, and w_z, u_z, u_r, b_z, and b_r are learnable weight matrices and bias vectors. The GRU may detect long-term dependencies in sequential data by carefully updating and outputting data at each time step. Overall, the GRU equations enable the framework to actively update and forget data from the prior hidden state via the update and reset gates and ingest new information from the input sequence through potential candidate activation. This enables the model to capture long-term associations in sequential data without experiencing the vanishing gradient problem that can occur with ordinary RNNs.
- The CNN includes convolutional layers, an activation function, pooling layers, a fully connected layer, and a dropout layer. Convolutional layers employ filters to generate feature maps using the provided data. Convolutional layers contribute to lowering the input size and speeding up training while avoiding overfitting the model. The most typical sort of pooling layer is known as the max-pooling layer. The parametric equation provided below has been employed with the max-pooling method for determining the maximum value of the domain that the pooling kernels cover [32]:
  (8) $$P^{l(i,j)}=\max_{(j-1)W+1\leq t\leq jW}\left\{a^{l(i,j)}\right\}$$
  where the values $P^{l(i,j)}$ and $a^{l(i,j)}$ represent the width of the pooling layer and neuron’s activation value. The activation function enhances the model’s adaptability, which permits the linear transformation of an inseparable problem into a detachable one. The Rectified Linear Unit (ReLU) function’s inherent ability to produce values between 0 and 1 renders it useful as an activation function [32].
  (9) $$a^{l(i,j)}=f\left(y^{l(i,j)}\right)=\max\left\{0,\right\}$$
  where $a^{l(i,j)}$ denotes the activation value of the layer output $y^{l(i,j)}$. The previously retrieved information is combined with the fully connected layers to produce the final prediction, as shown in the following function [32]:
  (10) $$z^{l+j}=\sum_{i=1}^{n}\omega_{ij}^{l}a^{l(i)}+b_{j}^{l}$$
  where $\omega_{ij}^{l}a^{l(i)}$ and $b_{j}^{l}$ represent the bias values, the weight of the ith neuron, and the ith neuron of the lth layer given the input data length n.

#### 3.1.4. The Proposed Hybrid Models

**Figure 2:**
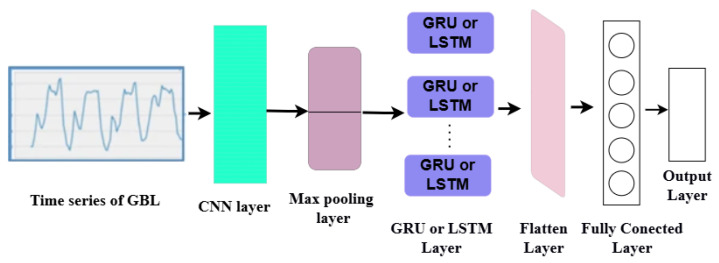
Proposed model for forecasting BGL.

The proposed hybrid models are based on a CNN and LSTM or GRU, as shown in Figure 2. The CNN extracts meaningful features from the input data and then uses GRU layers to capture temporal dependencies and sequence information based on these extracted features. The steps are as follows:

- The Conv1D layer receives a time-series vector. It works by convolving an input with a kernel size and filter size to produce feature maps and uses ReLU as an activation function to introduce non-linearity.
- The max-pooling layer selects the maximum value of the feature maps to reduce the spatial dimensions.
- The GRU or LSTM layer receives the output of the max-pooling layer to capture temporal dependencies and sequence information. It includes the number of ReLUs as an activation function.
- The flattened layer converts the multi-dimensional array into a one-dimensional array.
- The fully connected layer receives the flattened vector, which includes the number of united ReLUs as an activation function.
- The output layer generates the final output of forecasted BGL.

#### 3.1.5. Hyperparameter Optimization Methods

Optimization models are essential in improving a model’s performance and selecting the best parameters. KerasTuner is open-source software that optimizes hyperparameters in Keras models [33]. It offers an intuitive interface across various hyperparameter-tuning algorithms, making it straightforward to experiment using alternative optimization methods [33]. KerasTuner supports several commonly used hyperparameter optimization algorithms, including Bayesian optimization, which uses a Gaussian Process model to search the hyperparameter space [33] efficiently. Random search generates hyperparameter configurations at random from the search space.

Bayesian optimization is a popular method for hyperparameter optimization in machine learning. It is especially effective when the hyperparameter search space is complex, assessing a hyperparameter configuration is computationally costly, or the objective function is stochastic or non-convex [34]. The basic principle of Bayesian optimization is to generate a probabilistic model that resembles the underlying objective function, typically in the form of a Gaussian Process (GP) [34]. The GP model is employed to direct the search for the subsequent hyperparameter configuration to be assessed to identify the global optimum of the objective function as quickly as possible [35]. While determining the next hyperparameter combination, Bayesian optimization takes into consideration the previous evaluation findings and uses a probabilistic function to select a combination of parameters [35]. We define a set of values for hyperparameters in Table 2.

#### 3.1.6. Evaluating the Models

We used two methods to evaluate the models: Root Mean Square Error (RMSE), and Mean Absolute Error (MAE)

(11) $$\mathrm{RMSE}=\sqrt{\frac{1}{n}\sum_{i=1}^{n}{\left(y_{i}^{\mathrm{obs}\;}-y_{i}^{\mathrm{pred}\;}\right)}^{2}}$$

(12) $$\mathrm{MAE}=\frac{1}{n}\sum_{i=1}^{n}\left|y_{i}^{\mathrm{obs}\;}-y_{i}^{\mathrm{pred}\;}\right|$$

### 3.2. Online Forecasting Pipelines

The online phase aims to collect, process, analyze, and predict mortality in real-time. It was developed based on Apache Kafka, which uses big data stream management to store data, and Apache Spark, which processes and predicts health attributes.

#### 3.2.1. Big Data Platforms

We used Apache Spark and Apache Kafka to develop online prediction pipelines:

- Apache Spark is an open-source platform developed for processing big data and is particularly suited for iterative machine-learning applications. Spark is distinguished by its capacity for in-memory computations. It enables the data to be cached in memory, removing Hadoop’s restriction on disk overhead for iterative processes [36]. Spark is faster than previous methods for some jobs when the data can fit in memory and be stored on a disk. Spark is an engine that supports Java, Scala, and Python and operates on various systems. It can also run on the Hadoop management system and read data from HDFS.
  – Spark SQL is a Spark Core component that introduces Schema RDD, a modern data abstraction that supports structured and semi-structured data [36].
  – Spark Streaming is built on the Spark API for live data processing from sources such as Twitter and Kafka. With Spark batch engines, data streams are categorized into batches of less than a second interval and subsequently processed. Spark’s streaming module uses an extension called discretized stream, which consists of a sequence of mini-batches, each represented by a Spark RDD [36].
- Apache Kafka offers a performance-optimized binary TCP-based protocol and a message set concept that intelligently groups messages to save network hop costs [37]. It is a highly available, fault-tolerant cluster that spans several computers and data centers. It processes records as they are created in real-time. Developers use Producer, Consumer, Streams, and Connector APIs to publish, process, analyze, aggregate, convert, and integrate data sources [37]. Kafka is employed efficiently in real-time streaming data pipelines that reliably transmit data or event records between corporate systems at high rates without the danger of data corruption or duplication [37]. Likewise, apps use event record streams to support real-time streaming. In combination with various other Apache technologies, Kafka is frequently used as part of more extensive solutions for stream processing, event-driven architecture, and big data analytics.

#### 3.2.2. The Main Steps of Online Prediction Pipelines

Figure 1 shows the main steps of online prediction pipelines for BGL in real-time.

- Step 1: Sensor Setup
  We developed a Python script to generate a BGL streaming time series.
- Step 2: Data Ingestion
  We developed Python scripts to continuously generate time series of BGL and push them to the Kafka topic.
- Step 3: Data Preprocessing
  An instance of SparkContext is generated to use Spark Streaming capabilities. Spark Streaming reads streaming data from Kafka and cleans, transforms, and extracts the health attributes from these streaming data.
- Step 4: Online Prediction
  CNN-GRU is applied to the BGL time series, and online prediction is performed.
- Step 5: Data Storage
  The results are stored in databases or file storage systems for future reference and analysis.
- Step 6: Results Visualization
  Healthcare professionals interact with the system through web dashboards and mobile apps, allowing them to monitor and make decisions based on real-time data.

## 4. Results and Discussion

### 4.1. Experimental Setup

The LSTM, GRU, CNN, and hybrid models were implemented in Python 3 using Anaconda. The keras-tuner optimization technique was used to optimize the DL models; the values of each model parameter that was selected by it will be presented in each result subsection. Some values of model parameters were adapted: batch size: 1; number of epochs: 20. The time-series dataset was split into a 75% training set and a 25% testing set using different numbers of minutes in the past 3, 10, 13, 18, 20, 30 for forecasting the number of minutes in the future 5, 15, 20, 25, 30, and 60, respectively.

The models were evaluated using RMSE and MAE. The results of each forecast are presented in the following subsections.

### 4.2. The Results of Forecasting 5 Min

Table 3 presents the performance metrics of different models to forecast 5 min of BGL. The GRU and LSTM models have similar performance, with small differences in RMS and MAE values; GRU has the lowest RMSE at 2.7871. The CNN had slightly better performance than GRU and LSTM, with 2.7005 for RMSE and 2.2214 for MAE. CNNs have a proven ability to identify local patterns and structures to extract spatial features from the data, which is critical for tasks such as sequence analysis. Thus, CNNs can capture local dependencies more clearly than GRU and LSTM models. The GRU and LSTM models show similar performance, with slightly different RMSE and MAE values. GRU has a marginally lower RMSE, while LSTM has a higher MAE. The hybrid models significantly outperformed the single models. CNN-GRU had the best performance, with an RMSE of 1.5416 and MAE of 1.1384. Therefore, the CNN works on the input data until hierarchical features are extracted and learned. Then, the outputs of this stage are passed to the GRU layer, enhancing the ability to focus on higher-level abstractions and thus capture complex temporal relationships more efficiently. That is, the combination of the two architectures will lead to a significant improvement in performance in tasks that require feature extraction and sequence learning.

### 4.3. The Results of Forecasting 15 Min

Table 4 presents the performance metrics of various models for forecasting BGL 15 min into the future. It is observed that LSTM has the highest RMSE and MAE compared to other models at 2.8823 and 2.3444, suggesting that it has the least accurate performance. As a result, if the LSTM model is fed very long or short sequences, its performance is negatively affected because LSTM models are originally designed to capture long-term dependencies. The CNN had a slightly better performance than GRU and LSTM, with 2.3267 for RMSE and 1.8463 for MAE. CNNs are very effective in capturing local patterns and features in data due to the convolutional structure of their layers. In addition, the pooling layers of CNNs help directly reduce the dimensionality, thus preventing overfitting. The hybrid models significantly outperformed the single models. CNN-GRU had the best performance at 2.1182 for RMSE and 1.6369 for MAE. For the same reason mentioned for 5 min forecasting, with 10 min forecasting, the composition of the CNN with GRU proves to be efficacious by combining their advantages in one model. This hybrid model shows the best performance, with the lowest error rate, indicating the highest accuracy among the listed models.

### 4.4. The Results of Forecasting 20 Min

Table 5 presents the performance metrics of different models when forecasting BGL at 20 min and the best values of the model’s parameters. LSTM has the highest RMSE and MAE compared to other models at 2.8837 and 2.3450, indicating that it has the least accurate performance. LSTM’s complex gating structure, compared with both GRUs and CNNs, can hinder the desired performance. The CNN performed better than GRU and LSTM, with 2.5881 for RMSE and 2.0646 for MAE. The combination of several factors favoring the CNN, such as stable training dynamics, appropriate hyperparameter settings, and its known ability to extract and leverage local features effectively, contributes to its improved performance compared to the GRU and LSTM models. The CNN-GRU hybrid model significantly outperformed the single models. The CNN-GRU had the best performance at 2.3681 for RMSE and 1.9027 for MAE. What was observed in the previous tables is repeated in this table, where the combination of the CNN with GRU or LSTM outperformed the single models. This is because their combination can mitigate the overfitting risks and reduce the input data’s dimensionality.

### 4.5. The Results of Forecasting 25 Min

Table 6 presents the performance metrics of various models used to forecast BGL 25 min into the future and the best values of the model’s parameters. LSTM has the highest RMSE and MAE compared to other models at 2.8964 for RMSE and 2.3542 for MAE, indicating that it has the least accurate performance. The CNN performed better than GRU and LSTM, with an RMSE of 2.6164 and an MAE of 2.0836. The hybrid models significantly outperformed the single models. CNN-GRU had the best performance at 2.4614 for RMSE and 1.9806 for MAE. What was observed in the previous tables is repeated in the following table, where the combination of the CNN with GRU or LSTM was able to outperform the single models. This is because their combination can mitigate the risks of overfitting and reduce the input data’s dimensionality, paving the way for GRU and LSTM networks to play their roles by focusing on learning meaningful temporal patterns.

### 4.6. The Results of Forecasting 30 Min

Table 7 presents the performance metrics of different models for forecasting BGL at 30 min and the best values of the model’s parameters. LSTM has the highest RMSE and MAE compared to other models at 2.793 and 2.252, indicating that it has the least accurate performance. The CNN performed better than GRU and LSTM, with an RMSE of 2.739 and an MAE of 2.174. CNNs’ well-known efficiency in extracting local features depends on their convolutional layers, which can capture local features. With stable training dynamics, CNNs lead to better performance than LSTM or GRU. The hybrid models significantly outperformed the single models. CNN-GRU had the best performance at 2.5428 for RMSE and 2.0268 for MAE. Both the CNN-GRU and CNN-LSTM hybrid models leverage the strengths of both architectures. The process starts with the CNN, which efficiently handles local feature extraction, while GRU and LSTM handle sequential dependencies, resulting in improved overall performance compared to standalone models.

### 4.7. The Results of Forecasting 60 Min

Table 8 presents the performance metrics of different models for forecasting BGL at 60 min and the best values of the model’s parameters. LSTM and GRU models have approximately the same RMSE and MAE compared to other models at 2.7837 for RMSE and 2.2472 for MAE, indicating that they have the least accurate performance. The CNN performed better than GRU and LSTM, with an RMSE of 2.6759 and an MAE of 2.1417. The hybrid models significantly outperformed the single models. CNN-GRU had the best performance at 2.5763 for RMSE and 2.1094 for MAE.

### 4.8. Discussion

**Figure 3:**
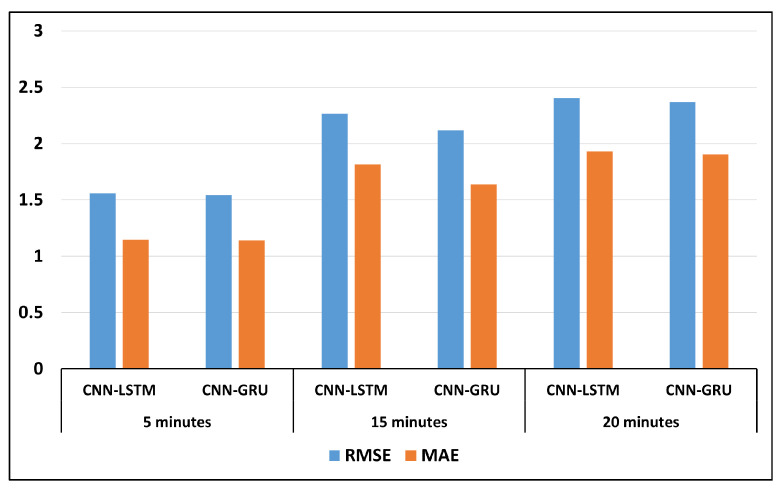
Comparing hybrid models for forecasting 5 min, 15 min, and 20 min.

**Figure 4:**
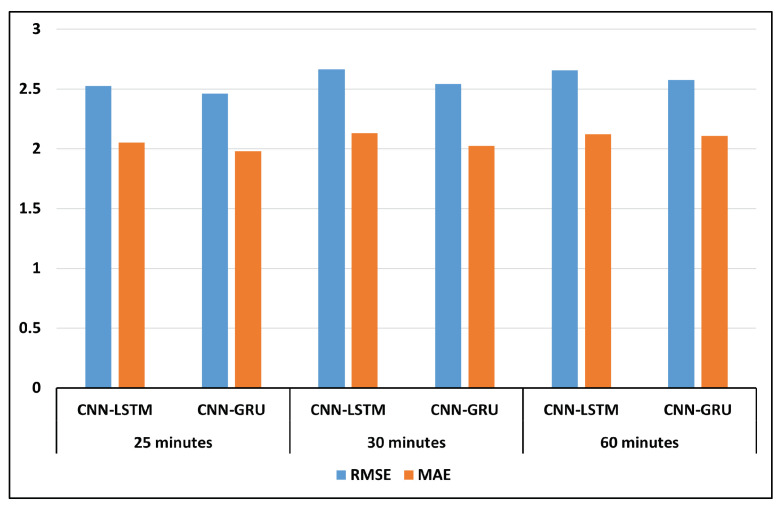
Comparing hybrid models for forecasting 25 min, 30 min, and 60 min.

We conducted various experiments to evaluate the proposed models, utilizing different lags and forecasting times. Figure 3 presents the performance metrics (RMSE and MAE) of two hybrid models (CNN-LSTM and CNN-GRU) evaluated over different forecasting times: 5 min, 15 min, and 20 min. The CNN-GRU model had the best performance, with lower RMSE and MAE compared to the CNN-LSTM model. Figure 4 presents the performance metrics (RMSE and MAE) of the two hybrid models (CNN-LSTM and CNN-GRU) evaluated over different forecasting periods: 25 min, 30 min, and 60 min.

### 4.9. Comparison with the State of the Art

Table 9 provides a comparison DL models with the proposed model for forecasting BGL on a type 1 diabetes dataset, using RMSE and MAE as performance metrics. The comparison also incorporates big data streaming platforms. The hybrid model based on CNN and GRU performs best because CNNs are highly effective at extracting local features and patterns from data. GRUs capture temporal dependencies in data more effectively over longer sequences. Combining a CNN with GRUs allows the model to extract meaningful features from the input data using the CNN layers and then use the GRU layers to capture temporal dependencies and sequence information based on these extracted features. In [20], the authors used Seq-to-Seq LSTM and obtained an RMSE of 17.52 and an MAE of 10.91. In [13], an RMSE of 18.930 at 30 min was obtained using a DL framework. In [14], a DRNN was applied and obtained an RMSE of 22.9 in 30 min. In [15], an RNN was used, which resulted in an RMSE of 15.95. In addition, previous studies have not forecasted BGL in real-time using big data platforms.

## 5. Conclusions

Accurately forecasting BGL poses a significant challenge for people living with diabetes, as the condition affects the body’s ability to metabolize food properly. This paper presents a hybrid model combining a CNN and GRU to enhance IoT-based diabetes management. The proposed hybrid CNN-GRU model for real-time blood glucose forecasting can extract meaningful features from the input data using the CNN layers and then utilize the GRU layers to capture the temporal dependencies and sequence information based on these extracted features. A publicly accessible BGL time-series dataset was converted to data for supervised learning based on different lags (window sizes) and different forecasting steps ahead. It was then scaled to 0 and 1 to help the DL model efficiently learn. The results show that the hybrid models had significantly enhanced performance compared to the single models. Personalized diabetes management, care task automation, and better decision assistance for medical personnel are all made possible by the suggested hybrid CNN-GRU model, which improves real-time blood glucose predictions for continuous monitoring.

## Figures and Tables

**Table 1:** A sample of the BGL time-series dataset.

Time	BGL
11/2/2020 20:23	8.9
11/3/2020 19:53	6.5
11/3/2020 20:08	7.4
11/3/2020 20:23	7.8
11/3/2020 20:39	8.2
11/3/2020 20:54	8.7
11/3/2020 21:08	9.4
11/3/2020 21:23	8.3
11/3/2020 21:38	7.4
11/3/2020 21:55	7.1
11/3/2020 22:10	8.3
11/3/2020 22:25	9.5

**Table 2:** Defining a set of values for hyperparameters.

Models	# Units (No. Memory Cells)	Dropout Value	Reg_Rate	Filter	Kernel
GRU	50	Range from 0.1 to 0.9	[0.01, 0.05, 0.005, 0.001]	-	-
LSTM	70	Range from 0.1 to 0.9	[0.01, 0.05, 0.005, 0.001]	-	-
CNN	170	Range from 0.1 to 0.9	[0.01, 0.05, 0.005, 0.001]	[200, 128, 250]	[3, 4]
CNN-LSTM	From 50 to 500 step 10	Range from 0.1 to 0.9	[0.01, 0.05, 0.005, 0.001]	[200, 128, 250]	[3, 4]
CNN-GRU	From 50 to 500 step 10	Range from 0.1 to 0.9	[0.01, 0.05, 0.005, 0.001]	[200, 128, 250]	[3, 4]

**Table 3:** The results of forecasting 5 min.

Models	RMSE	MAE	# Units	Dropout	Reg_Rate	Filter	Kernel
GRU	2.7871	2.2468	500	0.4	0.01	-	-
LSTM	2.78254	2.25403	90	0.4	0.1	-	-
CNN	2.7055	2.2214	110	0.4	0.001	200	4
CNN-LSTM	1.5601	1.1473	[160, 150]	02	0.005	128	3
CNN-GRU	1.5416	1.1384	[150, 140]	0.3	0.001	128	3

**Table 4:** The results of forecasting 15 min.

Models	RMSE	MAE	# Units	Dropout	Reg_Rate	Filter	Kernel
GRU	2.7823	2.2422	50	0.3	0.01	-	-
LSTM	2.8823	2.344	70	03	0.05	-	-
CNN	2.3267	1.8463	170	0.2	0.01	200	4
CNN-LSTM	2.2638	1.8152	[200, 50]	0.3	0.005	128	4
CNN-GRU	2.1182	1.6369	[150, 40]	0.2	0.001	128	3

**Table 5:** The results of forecasting 20 min.

Models	RMSE	MAE	# Units	Dropout	Reg_Rate	Filter	Kernel
GRU	2.7826	2.2495	70	0.3	0.1	-	-
LSTM	2.8837	2.3450	100	0.2	0.01	-	-
CNN	2.5881	2.0646	200	0.2	0.006	128	4
CNN-LSTM	2.4037	1.9306	[130, 100]	0.4	0.006	250	3
CNN-GRU	2.3681	1.9027	[140, 80]	0.2	0.1	200	3

**Table 6:** The results of forecasting 25 min.

Models	RMSE	MAE	# Units	Dropout	Reg_Rate	Filter	Kernel
GRU	2.7873	2.2522	70	0.3	0.1	-	-
LSTM	2.8964	2.3542	100	0.2	0.01	-	-
CNN	2.6164	2.0836	200	0.2	0.006	128	4
CNN-LSTM	2.5259	2.0515	[130, 100]	0.4	0.006	250	3
CNN-GRU	2.4614	1.9806	[140, 80]	0.2	0.1	200	3

**Table 7:** The results of forecasting 30 min.

Models	RMSE	MAE	# Units	Dropout	Reg_Rate	Filter	Kernel
GRU	2.7881	2.241	150	0.3	0.01	-	-
LSTM	2.793	2.252	250	0.4	0.05	-	-
CNN	2.739	2.174	150	0.3	0.001	200	3
CNN-LSTM	2.6632	2.1324	[128, 100]	0.4	0.001	128	4
CNN-GRU	2.5428	2.0268	[180, 150]	0.2	0.0001	200	4

**Table 8:** The results of forecasting 60 min.

Models	RMSE	MAE	# Units	Dropout	Reg_Rate	Filter	Kernel
GRU	2.7842	2.2458	190	0.3	0.1	-	-
LSTM	2.7837	2.2472	200	0.3	0.05	-	-
CNN	2.6759	2.1417	250	0.2	0.005	128	3
CNN-LSTM	2.6574	2.1227	[180, 130]	0.4	0.006	200	3
CNN-GRU	2.5763	2.1094	[200, 160]	0.2	0.1	200	3

**Table 9:** Comparison with the state of the art.

Papers	Models	Datasets	Big Data Streaming Platforms	Result
[20]	Seq-to-Seq LSTM	OhioT1DM (Type 1 Diabetes)	No	RMSE 17.52 MAE 10.91
[13]	DL learning framework	OhioT1DM (Type 1 Diabetes)	No	30 min: RMSE = 18.930
[14]	DRNN	OhioT1DM (Type 1 Diabetes)	No	30 min: RMSE = 22.9
[15]	RNN	Ohio T1DM (Type 1 Diabetes)	No	30 min: RMSE = 15.95
[17]	Stacked regression	OhioT1DM (Type 1 Diabetes)	No	30 min: RMSE = 18.99 60 min: RMSE = 33.39
[21]	LSTM + RNN	Ohio T1DM (Type 1 Diabetes)	No	30 min: RMSE = 6.45 60 min: RMSE = 17.24
[38]	Fuzzy with PSO	OhioT1DM (Type 1 Diabetes)	No	RMSE: 22.3723
Our work	CNN-GRU	Kaggle (Type 1 Diabetes)	yes	RMSE = 2.3681 MAE = 1.9027

## Data Availability

All datasets used to support the findings of this study are available from the direct link in the dataset citations.
